# The Matron's Department

**Published:** 1905-12-09

**Authors:** 


### THE MATRON'S DEPARTMENT.

### XV.—GENERAL SUPERINTENDENCE.

The brief examination which has been attempted into some of the matron's duties in institutions will suffice to show that by far the most important of them all is the duty of educating subordinates to work equally well in her presence or absence. The matron cannot be everywhere at once, nor should she ever be reduced to wishing this were possible. Never for a moment should she allow herself to say, "This thing is never well done unless I do it myself." The pleasure of displaying her own talents, whether in nursing or in household matters, is one which she must continually deny herself. For should she linger in the enjoyment of personally undertaking someone else's work, a hundred matters of importance will be waiting for her attention, and some must perforce be neglected. Wherever possible, then, she must choose to work through instruments, and the recognition of this fact is the first step towards administrative success. It is not an easy step. The impulse with an energetic woman is always to do things herself—with her hands, that is to say, instead of with her head. But she must for ever be on her guard against burdening herself with duties because she finds this course less irksome than exacting their due performance from someone else. If she is to hold complete control over the complicated mass of details which go to make up a large household she must devote by far the larger proportion of her time to the planning of work for others, its inspection when carried out, the rectification of mistakes, and the supervison of those to whom the work is entrusted. It may be objected that all this minute attention to detail is more properly housekeeper's than matron's work. In a large institution the matron may, it is true, have a housekeeper to relieve her of much work which would otherwise leave her no time for other duties. But it is the matron who must be the mainspring of authority in household management whether she governs through a housekeeper or in person. It will be the housekeeper's duty, for instance, to prepare the daily requisition sheet of provisions, but the matron must have it submitted to her and go through it item by item. It is the housekeeper who will give out stores and keep the key of the store-room, but it will be open to the matron's inspection, and the better the housekeeper the more she will value a visit from her chief in her own department. The lists of all kinds which it will be the duty of the housekeeper to prepare will gain all their point from being submitted to the matron's eye. Her steady attention to household details will be reflected in the zeal of those who work under her, but if she is indifferent, always "too busy" to look through the reports prepared for her with much expenditure of labour, and content to trust everything to the housekeeper, that functionary will insensibly grow also to think her duties of only secondary importance.

As with the ward sisters, so with the housekeeper, assistant matron, and home sister, the matron must give her whole attention to each for the time being, enter into their difficulties, tell them frankly and in a business-like manner when things are going wrong, and let them perceive that good work is appreciated. Above all, she must invite their confidence by never betraying it.

It is important that the matron, if, as so often happens, she is single-handed, should train one or two of her sisters to undertake her duties in case of illness or emergency. One sister may be initiated into the office routine and get a little practice in answering business letters or making up the accounts. Another should be familiar with the duties of home sister, and a third should understand how to give out the stores, attend to the linen, and make out requisitions for the cook. All this would be of the greatest value to the sisters themselves, and would ensure matters running smoothly should the matron for any cause be suddenly absent.

In apportioning her time, the matron's main difficulty will be to avoid concentrating too much attention on one section of her household to the neglect of another. The patients, the nurses and probationers, the domestic staff, the medical officers, the governing body, and the public, all have claims on her time, and so to contrive that all have their due is a problem which the wisest woman can never hope to solve with complete satisfaction to herself. The best way to approach it is undoubtedly to come to a clear understanding as to what can and what cannot be attempted. In the first place, while recognising that the hospital exists for the patients and that she is responsible for their proper tendance, the matron must renounce, as has been already pointed out, any intimate share in the nursing. It is undesirable, even in a small hospital, that she should give herself up to the personal care of a bad case, or allow it to be felt that only in her presence is there safety. Such dependence is bad for the sister, bad for the general welfare of the hospital, and doubly bad for the matron herself in mind and body. Yet it is quite possible, even among some hundreds of occupied beds, for the matron to follow, day by day, the course of events in each ward as she visits it twice daily, to watch the progress of particular forms of treatment, and keep a keen eye on the due carrying out of details. It is unfortunately true that in some hospitals where omission would be least suspected, the treatment ordered by the physician in charge of the case is by no means inevitably followed out. Plasters are prescribed which the sister never finds time to apply, medicines are administered irregularly, and special diets altogether omitted. Incessant supervision on the part of the matron is needed to obviate any possibility of such neglect. The strength of a chain is the weakest link, and that must indeed be a fortunate institution in which blind confidence can be reposed in every ward-sister. It is not everyone who possesses the wonderful gift for remembering faces and facts which enables some noted matrons of the day to individualise every patient, but the more closely the head of the household can bring herself into touch with the patients as suffering human beings, instead of cases, the less likely is it that her visits to the ward will degenerate into interference or become mere mechanical routine.

The demands which the nurses and probationers make upon the matron's time have already been fully considered. In a training school of any size she cannot hope to exercise a great personal influence among them. She must perforce leave their shaping in other hands and will be more felt as a reserve force in the background than if she should attempt individual admonitions. In a small household danger lies in undue intimacy or familiarity, but the close association forced on the inmates of small institutions, if it creates special difficulties for the one in authority, affords at least a favourable opportunity for moulding character.

There should be fixed periods in the day when the matron is accessible in her office to members of the medical staff and of the governing board, and in proportion as these times are steadily adhered to will the waste of energy be avoided which is entailed when other duties have to be suddenly abandoned to attend to some urgent summons. Nothing more quickly makes itself felt than regularity, and the matron must be prepared for the necessity of gently educating, by her own punctual observance of routine, colleagues who may be even busier than herself yet less businesslike.

In some hospitals the public make rather large demands on the matron's time. There is no doubt that much outside support can be attracted if the hospital can be made accessible to visitors without interfering with more important work, and the only way of doing this is to turn goodwill into a practical channel, instead of allowing it to evaporate in talk. The matron will find the merely curious and idle easily discouraged if she can make it felt that every visit entails some practical service. It is only the genuine worker who likes to be greeted with a cordial welcome because there is some lame child just being sent out of hospital whose after-care is to be undertaken, or warm clothing to be put in hand for Christmas. Regular visitors to the wards should have more attention than is often accorded to them. Their names should be submitted to the weekly board for sanction, and their hours and wards formally assigned to them after consultation with the matron. Her advice will be generally accepted with gratitude as to the disposition of their time, and the ward sister should be encouraged to bring under their notice any patients likely to appreciate their visits. The matron must not be too timid to limit the length of visit or interpose if any tactless visitor prove an interruption to the work. The visitors, in fact, constitute a body of workers, probably untrained, but certainly full of zeal, and it is for the matron to extract the best work from them which they can be made to give. If the training of them seems to her at times to add a new and wholly unnecessary burden to her shoulders, she must also remember that every skilled visitor she can enlist in the service of the patients will in times of perplexity be ready to solve her difficulties and bring her outside help.

